# Molecular docking based screening analysis of GSK3B

**DOI:** 10.6026/97320630015201

**Published:** 2019-03-15

**Authors:** Adewale J Ogunleye, Afeez J Olanrewaju, Michael Arowosegbe, Olaposi I Omotuyi

**Affiliations:** 1 Centre for Biocomputing and Drug Discovery, Adekunle Ajasin University, Nigeria; 2 BIOTRUST BIOTRUST Scientific, Nigeria, Nigeria; 3 Department of Anatomy, Babcock University; 4 Lagos State University, Ojo, Lagos Nigeria

**Keywords:** Alzheimer's diseases, invasive ductal breast carcinoma, ductal breast carcinoma in-situ, GSK-3B, molecular docking, oncomine

## Abstract

GSK3B has been an interesting drug target in the pharmaceutical industry. Its dysfunctional expression has prognostic significance in the top 3 cause of death associated with non-communicable diseases (cancer, Alzheimer's disease and type 2 diabetes). Previous studies have shown clearly that inhibiting GSK3B has proven therapeutic significance in Alzheimer's disease, but its contribution to various cancers has not been clearly resolved. In this study we report the contribution and prognostic significance of GSK3B to two breast cancer subtypes; ductal carcinoma in-situ (DCIS) and invasive ductal carcinoma (IDC) using the Oncomine platform. We performed high throughput screening using molecular docking. We identified BT-000775, a compound that was subjected to further computational hit optimization protocols. Through computational predictions, BT-000775 is a highly selective GSK3B inhibitor, with superior binding affinity and robust ADME profiles suitable for the patho-physiological presentations.

## Background

Alzheimer disease (AD) is a neurodegenerative disorder which happens to be the most common cause of age-related dementia currently accounting for up to 70% of all dementia cases based on clinical diagnostic criteria [1]. The unprecedented level of aging in developed nations is leading to an increase in the burden of AD and it has been linked to an estimated health-cost of about US$ 172 billion per year in the world [2]. The World Alzheimer Report 2010 evaluated that ageing of the global population will aggravate economic effect of dementia more than that of cancer, heart disease, and stroke combined [3-4] and that by 2050, an estimated 14-16 million people in the U.S. alone will be diagnosed with the disease if novel treatments to prevent or delay the onset of AD are not identified [5].

AD is categorized by a continuous loss of episodic memory and by cognitive and behavioural impairments [6]. The most important histo pathological hallmarks of AD are extracellular senile plaques composed by amyloid-β (Aβ) protein and neurofibrillary tangles (NFTs)-formed mainly by GSK3B mediated hyper phosphorylation of tau proteins [7]. GSK3β is the most important tau kinase in neurons and the phosphorylation state of tau determines its ability to stabilize microtubules [7]. In experimental models of AD, GSK3β has been shown to cause hyper phosphorylation of Tau, leading to microtubule disassembly and loss of neuronal function [8]. In addition, the activation of GSK3β inhibits the secretory cleavage of the amyloid precursor protein (APP), elevating the production of the Aβ peptide [9], thereby leading to memory impairment in animal models. Thus, the dysregulation of GSK3β activity has crucial effects in key pathological features of AD and its atypical activation may be involved in the initial and primary event in the physiopathology of AD [10].

In parallel, GSK3β has previously been described as a key regulator due to its diverse cellular functions. GSK3β has been linked to tumorigenesis and the development of cancer as formally established by in vitro and in vivo experiments when dys-regulated [11]. A number of studies have revealed that GSK3β can positively regulate the proliferation and apoptosis of tumour cells [11]. In the same vein, more studies have demonstrated that the inhibition of GSK3β induces apoptosis in various types of cancers, such as colorectal cancer, pancreatic cancer and bladder cancer [12]. Nonetheless, Zeng and colleagues affirmed that the role of GSK3β differs in different cancers 12 and we wish to explore it as a viable target in breast cancer.

Breast cancer (BC) is extremely heterogeneous as regards its aetiology and pathophysiological characteristics. In fact, BC is the most commonly diagnosed cancer among women worldwide [13]. Epidemiological estimates have it that in 2012, about 1.7 million new cases of breast cancer, which accounted for 12% of all cancers or 25% of all female cancers that were, diagnosed [14]. Histologically, BC has been described in two major forms, in situ breast carcinoma and invasive (or infiltrating) breast carcinoma [15]. Breast carcinoma in situ is further sub-divided into ductal or lobular carcinoma in situ (DCIS or LCIS) [15]. DCIS has a higher prevalence over LCIS and due to its heterogeneity, has been further classified into comedo, cribi form, micro papillary, papillary and solid [14]. On the other hand invasive breast cancer has been classified into invasive ductal, lobular, mucinous, tubular, medullary and papillary breast carcinoma [16]. Similarly, invasive ductal carcinoma of the breast (IDC) accounts for 70-80% of all diagnosed cases of invasive breast cancers [17]. Despite the high prevalence of DCIS and IDC, and the abundance of molecular targets [13, 17], it is pretty difficult to say that there is an efficient drug molecule for their control.

Using potent bioinformatics tools, we identified GSK-3β as an overexpressed oncogene in DCIS and IDC samples. Using clinical data, we further evaluated and confirmed the overall prognostic dependency of breast cancer patients on GSK-3β. Using this backdrop, we sought to identify novel small molecule inhibitor of GSK-3β with the aim of halting the progression of AD, IDC and DCIS breast cancers. 

## Methodology

### Target Validation:

GSK3B is an already established target for clinical trials in AD [18] but not breast cancer (DCIS and IDC) therapy. Target validation for GSK3B as an oncogene was performed on the Oncomine platform [19]. The search term 'GSK3B' as a 'gene' was inputted in the search bar and the output was used to perform a cross-tissue, cross-sample and cross-dataset analysis of the expression pattern of GSK3B. Oncomine was instructed to compare cancer samples with normal tissue samples within different experimental datasets. The total output was filtered to present only cases where GSK3B was among the top 1% overexpressed genes, has a threshold fold change of 2.0 and a maximum p-value of 1.0E-4. Copy number variations (CNV) was also assessed on the same platform to study impacts of mutation on chromosomal hotspots bearing GSK3B [20]. The 'TCGA Breast 2 dataset' was used solely for this purpose. This was because of its higher histo-pathological resolution of breast cancer subtypes and CNV analysis. Heatmap data was exported from Oncomine and visualized using Prism 7 (GraphPad Inc.) 

### Survival Analysis:

The prognostic significance of GSK3B for DCIS and IDC breast cancers was determined here. In this case, Kaplan-Meier Plotter (km-plot), a web-based algorithm for the prediction of overall survival (OS) rates among cancer patients, through the integration of clinical and gene expression data [21]. On km-plot, clinical and gene expression data of lung, liver, ovarian, gastric, and breast cancers are available. With km-plot, it is possibly to resolve the relationship between proteins, drugs and cancer progression over time. With the purpose of assessing the prognostic value of a specific gene, the patient samples were divided into two cohorts according to the median expression of the gene (high vs. low expression) and correlated with time course (usually in months). Hazard Ratio (HR) and the log ranked p-value were computed. At the same time, overall survival rates (probability) were computed and plotted against time (months). 

### Molecular Docking:

PDB coordinate files of human (Homo sapiens) GSK3B bound to an inhibitor was retrieved from the protein data bank (PDB CODE: 5K5N) (ref) and prepared using the protein preparation wizard. Briefly, the OPLS2005 force field was used to prepare the protein and pH was set at 7.0±2.0. All steric clashes were corrected, hydrogen bond order was fixed while missing side chains and loops were corrected using Prime. Using the same pH, 1000 ligands (retrieved from LigandBox) [22] were force-field treated using OPLS2005 force field on Ligprep and readied for molecular docking. Prior to docking, a grid box located at (x = 24.926, y = 24.926, z = 27.613) with a length of 10.00å, which correlates with the ATP site of GSK3B was defined for ligand sampling. On glide, molecular docking was first performed with the HTVS algorithm, followed by SP and then XP algorithms. Compounds with suboptimal docking score i.e. less than the already existing inhibitors retrieved from RCSB PDB were dropped after each step.

### Pharmacological Studies:

Although the LigandBox database comprised only drug like molecules i.e. they passed the Lipinski rule of five [23], this is not enough to conclude on the pharmacological prudency of our hit compound (BT-000775) for use in the light of these two pathophysiology. Computational prediction of parameter that account for the absorption, distribution, metabolism and excretion of BT- 000775 were done using QikProp (Schrodinger Inc.).

## Results and Discussion

### GSK3B is a valid drug target for AD, DCIS and IDC Breast Cancer

**Figure 1:**
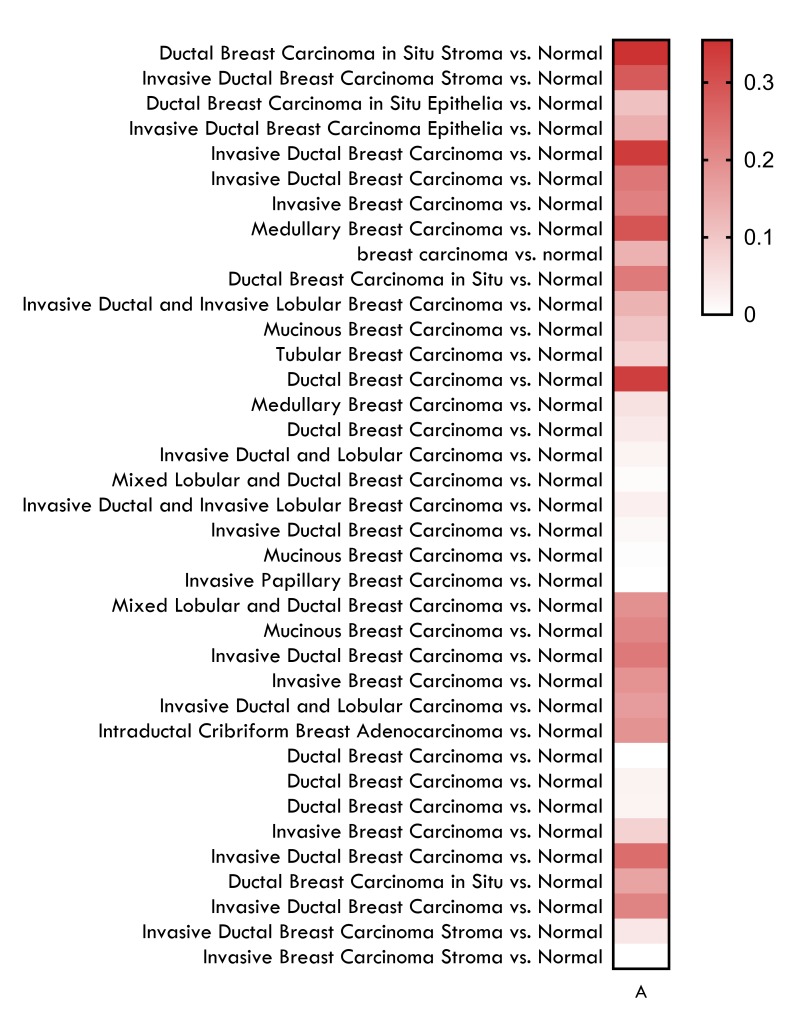
Heat map visualization of the fold change value for GSK3B expression 37 samples (top 1% over expressed). Heat map was plotted using the log(FC) coefficient after comparing normal breast tissue samples against DCIS and IDC samples.

A total of 14 datasets, which compared the expression of GSK3B in normal tissues with BC tissues, were retrieved from the Oncomine platform. These 14 datasets (representing Top 1% GSK3B overexpression) captured 57 clinical samples of breast cancer. Out of these 57 samples, 37 (64.9%) of them are DCIS (11) and IDC (26) samples (Table 1). These samples were isolated and submitted for analysis and visualization on Oncomine (Figure 1). A median ranked p-value of 1.78 x 10-7 was computed on Oncomine which shows significance even at 10E-3. Overall, there was an obvious over expression of GSK3B in DCIS (p-value = 5 x 10-2, LogFC = 1.21) and all the subtypes of IDC (p-value = 6.5 x 10-3, LogFC = 1.21) (as summarized on Breast Cancer (http://www.breastcancer.org)). On the average, mean fold change was found to be higher in the DCIS (LogFC = 1.42) samples as compared to IDC samples (LogFC = 1.42). This affirms that GSK3Bhas an altered expression pattern (up regulated) in DCIS and IDC subtypes of breast cancer.

**Figure 2:**
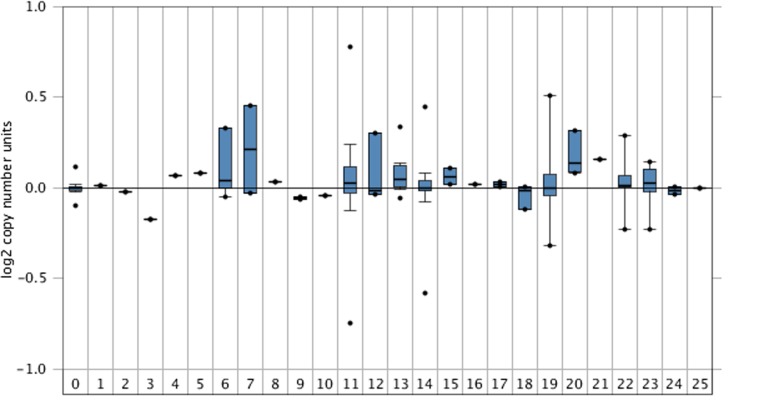
Pan Sample copy number variation analysis for GSK3B on 'TCGA Breast 2' using the Oncomine platform. 813 normal breast tissue samples were compared with 786 heterogenous samples.

Next, we assessed the copy number variations of GSK3B in its chromosomal location. CNV analysis was performed using the 'TCGA Breast 2' experimental dataset on Oncomine. This dataset was exclusively selected due to its higher pan-tissue resolution of breast cancer especially in the context of CNV [20, 24]. All major breast cancer types and subtypes were presented in the output plot represented in Figure 1. 813 normal breast tissue samples were compared with 789 BC samples (which captured 25 different patho physiological types of BC). The highest copy number variation was observed in IDC breast cancer type with 642 samples analysed while the highest median variation in copy number was accounted for by 6 DCIS samples. From the plot (Figure 2), IDC and DCIS samples presented a high discrepancy in the copy numbers as compared to control (normal) breast tissues. In the logarithm scale (to base 2), maximum CN-Variations was ±0.13 for normal breast tissues while CNVs that were as high as 0.75 and low as -0.8 were reported for IDC on Oncomine. This suggests that the chromosomal locus of GSK3B experiences a high level of both insertion and deletion mutation, and its effects are likely responsible for IDC metastasis. CNV box plots for DCIS were especially bound to the upper limits thereby suggesting that the genetic locus for GSK3B is exposed to addition/insertion mutations. This incremental variation in chromosomal copy number strongly suggests a yet uncovered mutagenetic role for the locus of GSK3B in the metastatic proliferation of DCIS and IDC cancer types and subtypes. In summary, we have created a premise based on literature, transcriptomic and genetic polymorphism evidences that GSK3B is a target for AD and DCIS/IDC breast cancer subtypes.

### GSK3B is a valid prognostic candidate for IDC and DCIS breast cancers

**Figure 3:**
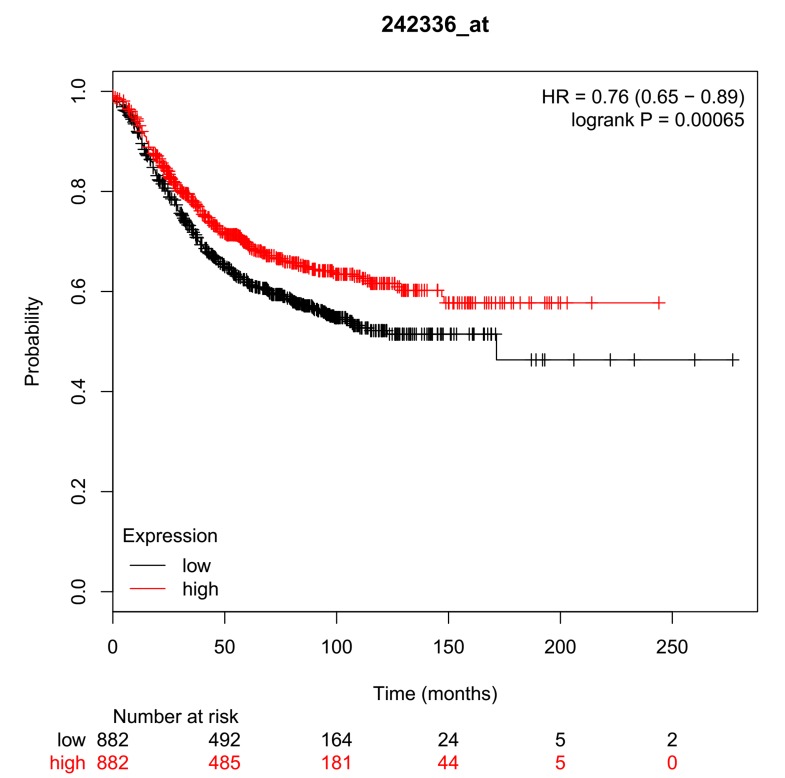
Survival curve for GSK3B (242336_at) in breast cancer tissue. This plot was made from kmplot.com. The black lines represent underexpression while the red lines represent overexpression. A p-value of 6.5e-4 was computed.

At this stage, we wanted to know if the over expression of GSK3B had any significance to BC prognosis and patient survival in the long run. If yes, this nullifies the chances that GSK3B over expression is as a result of other metabolic disorders 21, 25. Clinical data containing the patients' survival rate was parametrically integrated with their genomic data using the Kaplan-Meier plotter 21. Survival curves shown in Figure 3 (probability against time in months) showed that high expression levels of GSK3B correlates with short overall survival and that low expression of GSK3B correlates with increased survival rates in a significant fashion (pvalue=6.8 x 10-5,HR=0.76(0.65-0.89)). The computed median hazard ratio 0.76 implies a relatively low odd (or death risk) associated with the inhibition of GSK3B in previous breast cancer therapy. From the above, we have adjudged GSK3B to be of prognostic significance of GSK3B to BC and that it is not a result of some other underlying aberrations.

### Molecular docking identifies BT-000775 as a better small molecule inhibitor for GSK3B than previous inhibitors

Up next, we carried out a high throughput screening of 1000 drug like compounds (see Lipinski's rule of 5), which were made available through the Ligand Box database. These compounds were sampled iteratively within the GSK3B active (ATP) site (represented as a grid: see methods). A parallel molecular docking experiment of 25 GSK3B inhibitors (which were retrieved from RCSB PDB) was performed as comparative reference. In conventional laboratory practice, this is likened to the positive control. Interestingly, the binding affinity of BT-000775 (- 9.73Kcal/mol) was more favourable energetically when compared to the reference compounds (≤-8.47kcal/mol) (see Table 2).

**Figure 4:**
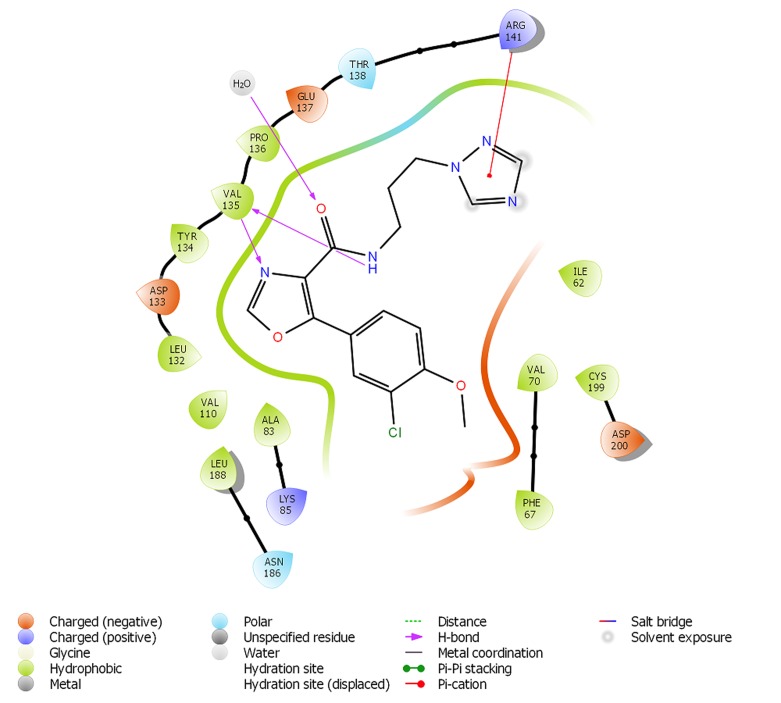
Receptor ligand interaction for PF357 within the GSK3B active site. The purple lines represent hydrogen bonds and their arrows signify the order of proton transfer. The red line represents the carion-p stacking.

**Figure 5:**
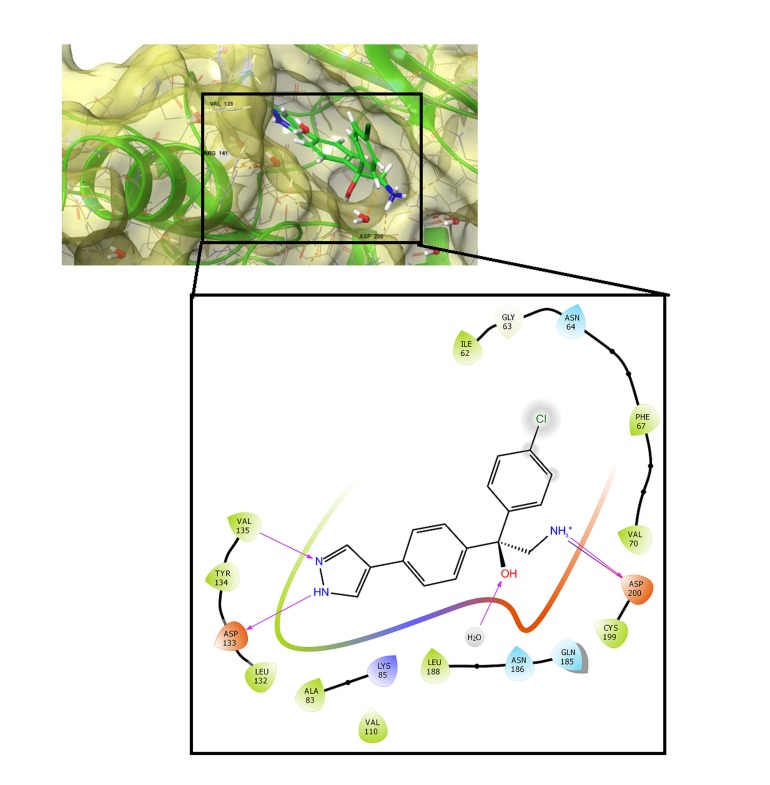
Ligand receptor for BT-000775 (Docking score = -9.73 kcal per mol) within the GSK3B active site. The purple lines represent hydrogen bonds and their arrows signify the order of proton transfer.

Liang and colleagues [26] carried out a comprehensive analysis of the chemistry of ligand-GSK3B interaction using PF-367, a highly selective GSK3B inhibitor that was reported in the crystal structure used in this study (5K5N). Similarly, Arfeen et al. carried out an intensive study on the specific residues that are crucial for GSK3B selectivity over other kinases with close homology [27]. In the works, proton transfer (hydrogen bond formation) and electrostatic interactions between two residues namely; Val 135 and Asp133 were highlighted for GSK3B active site inhibition. They also noted that p-cation stacking with Arg141 and 3'-Cl on the phenyl ring was enough to displace bound water molecule, thereby accounting for increased potency of the compound [26-27]. We implemented the power of Glide XP to predict the chemical interaction of GSK3B/PF-367 and GSK3B/BT-000775 using the ligand interaction diagram interface in Maestro (Schrodinger Inc.). The software effectively predicted the same binding of PF-367 to GSK3B as shown in Figure 4. For BT-000775, (Figure 5) the amide nitrogen on prayzole ring donates a proton to ASP 133 to form a hydrogen bond while Val 135 establishes a hydrogen bond with the nitrogen on the same ring. Meanwhile, the chlorine atom on the phenyl ring displaced water molecules away thereby disrupting the large hydration shell that spans Val62 to Val70. According to previous studies, it is believed that the disruption of this hydration shell must have has been linked to increased bioactivity of previous GSK3B inhibitors [26, 27]. In addition, the azanylium ion between the two phenyl rings established a strong hydrogen bond as well as a salt bridge with Asp200 while the hydroxyl group toform a water assisted hydrogen bond. Overall, we have shown that BT-000775 has a desirable energetic and chemical interaction profile when compared with previous inhibitors of GSK3B, a valid drug target for AD, DCIS and IDC using computational tools.

### Computational predictions show that BT-000775 has an excellent pharmacological profile

The pharmacological profile of BT-000775 as a small molecule inhibitor of GSK3B was ascertained in the light of the pathophysiology under study. We calculated several parameters that account for the absorption, distribution, metabolism and excretion (ADME) profile of BT-000775. These parameters are optimal for a neuro active compound targeting tauo pathies in the brain and onco-proteins in stromal and epithelial tissues (Table 3). For the computation of these parameters, QikProp a well-validated computational approach was implemented through Maestro (Schrodinger Inc.). The recommended range, which was provided on QikProp, was used to ascertain the optimality of each parameter. BT-000775 was predicted to have a normal activity on the central nervous system (CNS=0), an optimal blood brain barrier partition coefficient (QPLogBB = -0.573 QPPMDCK = 104.061), a high human oral absorption (Human Oral Absorption = 3) with only a very few fraction binding to human serum albumin (QPLogKhsa = 0.107). The above listed parameters account for the absorptive and distributive coefficients of BT-000775. It suggests that BT-000775 can be optimized for oral administration, and it is sure to pass the blood brain barrier with no hyperactivity on the neurons of the CNS. Metabolically, (#metab+ = 3) BT-000775 was predicted to be involved in number of metabolic reactions, which was still adjudged reasonable. Overall, these pharmacological metrics justify the suitability of BT-000775 as a pharmacologically sound compound for GSK3B-based therapy of AD, DCIS and IDC. (More information is available on the Schrodinger QikProp website)

## Conclusion

GSK3B is a known drug target for Alzheimer's disease and breastcancer. Its prognostic role has been confirmed in two breast cancer subtypes; invasive ductal breast carcinoma and ductal carcinoma in-situusing the Oncomine bioinformatics platform. BT- 000775 was further identified to be a highly selective inhibitor of GSK3B using molecular modelling based screening. The binding affinity, chemical interaction and ADME properties were further predicted computationally and comparedwith PF-367 (an investigational small molecule inhibitor of GSK3B). Results show that BT-000775 is a promising GSK-3B-inhibitor to be optimized as a lead compound in further pharmaceutical processes.

## Conflict of Interest

Authors declare no conflict of interest

## Figures and Tables

**Table 1:** Table showing GSK3B expression in 37 breast tissue samples (11 ductal breast carcinoma in situ (DCIS) and 26 invasive breast carcinoma (IDC)) out of 57 samples (64.9%) as retrieved from Oncomine.

S/N	Dataset	Sample No	Sample	P-value	Fold change
1	Ma Breast	1.	Ductal Breast Carcinoma in Situ Stroma vs. Normal	1.42E-07	2.263
		2.	Invasive Ductal Breast Carcinoma Stroma vs. Normal	6.23E-04	1.928
		3.	Ductal Breast Carcinoma in Situ Epithelia vs. Normal	0.008	1.279
		4.	Invasive Ductal Breast Carcinoma Epithelia vs. Normal	0.01	1.375
2	Zhao Breast	1.	Invasive Ductal Breast Carcinoma vs. Normal	6.65E-07	2.167
3	Curtis breast	1.	Invasive Ductal Breast Carcinoma vs. Normal	1.40E-80	1.724
		2.	Invasive Breast Carcinoma vs. Normal	3.88E-06	1.657
		3.	Medullary Breast Carcinoma vs. Normal	7.45E-10	1.973
		4.	breast carcinoma vs. normal	5.11E-05	1.357
		5.	Ductal Breast Carcinoma in Situ vs. Normal	1.00E-03	1.695
		6.	Invasive Ductal and Invasive Lobular Breast Carcinoma vs. Normal	4.46E-14	1.352
		7.	Mucinous Breast Carcinoma vs. Normal	1.78E-07	1.26
		8.	Tubular Breast Carcinoma vs. Normal	1.75E-06	1.193
4	Richardson Breast	1.	Ductal Breast Carcinoma vs. Normal	1.89E-07	2.157
5	TCGA Breast 2	1.	Medullary Breast Carcinoma vs. Normal	2.80E-02	1.125
		2.	Ductal Breast Carcinoma vs. Normal	9.30E-02	1.09
		3.	Invasive Ductal and Lobular Carcinoma vs. Normal	1.30E-02	1.045
		4.	Mixed Lobular and Ductal Breast Carcinoma vs. Normal	3.22E-01	1.015
		5.	Invasive Ductal and Invasive Lobular Breast Carcinoma vs. Normal	2.52E-01	1.063
		6.	Invasive Ductal Breast Carcinoma vs. Normal	9.46E-08	1.027
		7.	Mucinous Breast Carcinoma vs. Normal	4.21E-01	1.005
		8.	Invasive Papillary Breast Carcinoma vs. Normal	7.91E-01	-1.027
6	TCGA Breast	1.	Mixed Lobular and Ductal Breast Carcinoma vs. Normal	1.01E-04	1.556
		2.	Mucinous Breast Carcinoma vs. Normal	3.00E-03	1.615
		3.	Invasive Ductal Breast Carcinoma vs. Normal	1.91E-18	1.701
		4.	Invasive Breast Carcinoma vs. Normal	4.83E-10	1.538
		5.	Invasive Ductal and Lobular Carcinoma vs. Normal	2.00E-02	1.48
		6.	Intra ductal Cribri form Breast Adeno carcinoma vs. Normal	5.20E-02	1.533
7	Perou Breast	1.	Ductal Breast Carcinoma vs. Normal	7.10E-01	-1.185
8	Sorlie Breast	1.	Ductal Breast Carcinoma vs. Normal	4.38E-01	1.053
9	Sorlie Breast 2	1.	Ductal Breast Carcinoma vs. Normal	4.48E-01	1.048
10	Gluck Breast	1.	Invasive Breast Carcinoma vs. Normal	1.70E-02	1.193
11	Radvanyi Breast	1.	Invasive Ductal Breast Carcinoma vs. Normal	1.05E-01	1.782
		2.	Ductal Breast Carcinoma in Situ vs. Normal	2.04E-01	1.439
12	Trashvili Breast	1.	Invasive Ductal Breast Carcinoma vs. Normal	2.10E-01	1.637
13	Karnoub breast	1.	Invasive Ductal Breast Carcinoma Stroma vs. Normal	2.25E-01	1.102
14	Finak Breast	1.	Invasive Breast Carcinoma Stroma vs. Normal	1.00E+00	-2.224

**Table 2:** Table showing the docking scores of previously reported GSK3B inhibitors retrieved from protein databank and Drugbank (*).

Title	glide gscore
5K5N	-8.47
TIP002092*	-8.246
5352032*	-8.097
4NM0	-6.738
3ZRL	-5.859
3F88	-5.291
3ZRK	-5.439
3ZRK	-5.984
1Q4L	-5.07
4PTC	-6.031
3F7Z	-4.98
4IQ6	-4.956
3ZRL	-5.616
1Q5K	-4.778
4PTG	-5.606
3DU8	-5.089
5F94	-4.257
4PTG	-4.668
4PTG	-5.342
3Q3B	-4.245
4PTC	-4.41
3ZRK	-4.965
3I4B	-4.15
1Q3W	-3.562
3ZRM	-2.83
1Q3W	-3.566
3ZRM	-3.001

**Table 3:** Table showing the Absorption, Distribution, Metabolism and Excretion (ADME) profile of BT-000775. The full QikProp output and range is available in the supplementary file.

Title	BT-000775
docking score	-9.727
CNS	0
mol MW	313.786
donorHB	4
accptHB	3.25
QPpolrz	32.257
QPlogPC16	11.781
QPlogPoct	19.039
QPlogPw	12.145
QPlogPo/w	2.402
QPlogS	-2.986
CIQPlogS	-3.884
QPlogHERG	-6.262
QPPCaco	93.331
QPlogBB	-0.573
QPPMDCK	104.061
QPlogKp	-4.794
#metab	3
QPlogKhsa	0.107
HumanOralAbsorption	3
PercentHumanOralAbsorption	76.267
RuleOfFive	0
RuleOfThree	0
Jm	0.005
